# Supplementary material for: Oscillatory rheotaxis of artificial swimmers in microchannels
Source: Nat Commun. 2022 May 26;13:2952. doi: 10.1038/s41467-022-30611-1 (PMC9135748; doi:10.1038/s41467-022-30611-1)
Supplement: Supplementary file 1 — Supplementary Information [file 41467_2022_30611_MOESM1_ESM.pdf]

Supplemental Material: Oscillatory rheotaxis of artificial swimmers in microchannels

Ranabir Dey,[1,2,*] Carola M. Buness,[2] Babak Vajdi Hokmabad,[2] Chenyu Jin,[2,3] and Corinna C. Maass[2,4,†]

[1] *Department of Mechanical and Aerospace Engineering, Indian Institute of Technology Hyderabad, Kandi, Sangareddy, Telengana- 502285, India*
[2] *Dynamics of Complex Fluids, Max Planck Institute for Dynamics and Self-Organization, Am Faßberg 17, 37077 Göttingen, Germany and Institute for the Dynamics of Complex Systems, Georg August Universität Göttingen, Germany*
[3] *Physics Department, University of Bayreuth, 95440 Bayreuth, Germany*
[4] *Physics of Fluids Group, Max Planck Center for Complex Fluid Dynamics, MESA+ Institute and J. M. Burgers Center for Fluid Dynamics, University of Twente, PO Box 217,7500 AE Enschede, The Netherlands*

I. SUPPLEMENTAL VIDEOS

All videos have been sped up, which we have clarified by adding timestamps. Where applicable, the x coordinate is chosen to point upstream of the imposed flow. Oscillating trajectories are colour coded by $\langle v_x \rangle_{\Delta t}$, i.e. the x translation in the lab frame smoothed over a time interval Δt larger than the oscillation period, to give a better impression of the net upstream/downstream translation.

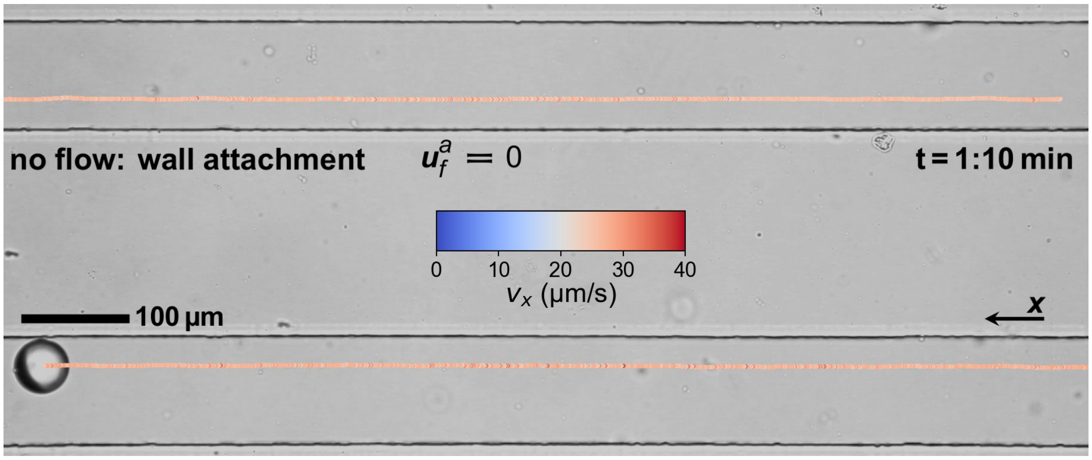

Supplemental Video S1: Active droplets in parallel channels without imposed flow. The droplets adhere to the channel side walls.

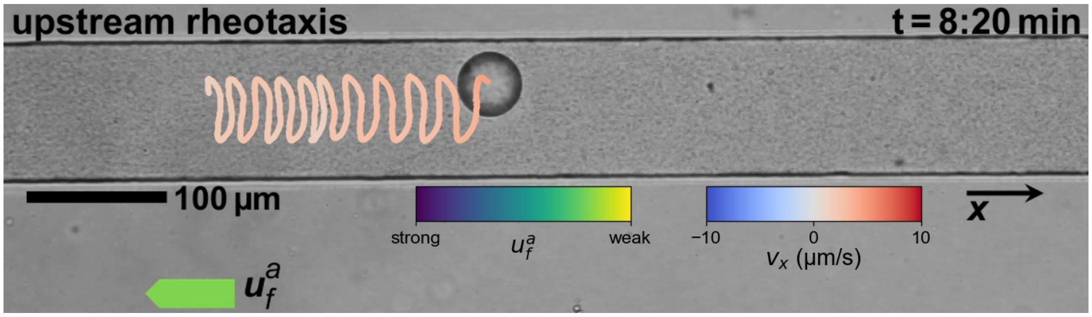

Supplemental Video S2: Control of rheotactic displacement from upstream oscillation over in-place trapping to downstream drift for a droplet by regulating the imposed flow.

* ranabir@mae.iith.ac.in
† c.c.maass@utwente.nl

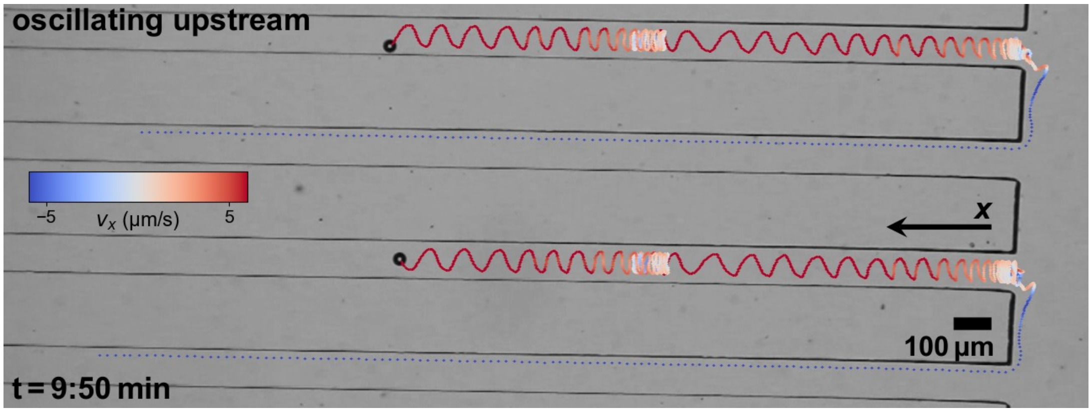

Supplemental Video S3: Example of two nearly synchronously oscillating droplets under similar flow conditions in two parallel channels. Initially, strong flow sweeps the droplets downstream at the walls; later, under weaker flow, and guided around corners, they are made to simultaneously travel upstream or are trapped in place by controlling the imposed flow.

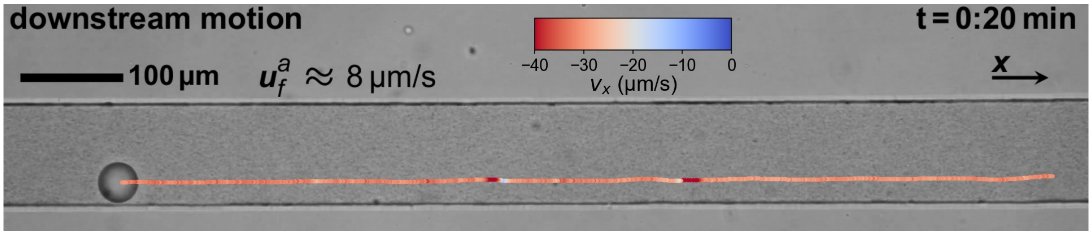

Supplemental Video S4: The rheotactic behaviour depends on the initial conditions. If the imposed flow starts in the droplet's swimming direction, *i.e.* $|\Psi(t=0)| \approx \pi$, the droplet will not switch direction to perform upstream oscillation, even if the flow speed would permit it - as in this case, where $\bar{u}_f^a \approx 0.3$.

II. SUPPLEMENTAL FIGURES

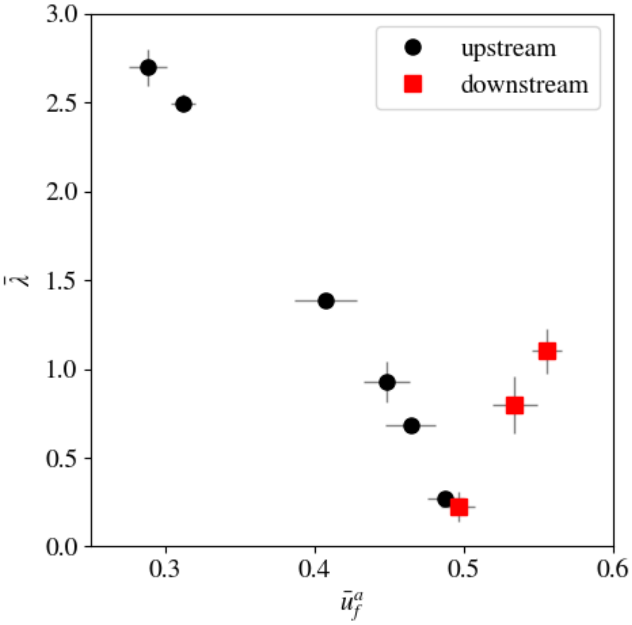

Supplemental Figure S1: Variation of the wavelength of the active droplet trajectory during the oscillatory upstream rheotaxis and the swinging downstream drift with increasing strength of the imposed pressure-driven flow. The non-dimensional wavelength ($\bar{\lambda} = \lambda/R_d$) of the oscillatory trajectory decreases with increasing $\bar{u}_f^a$ (black markers) during the upstream rheotaxis. However, during the swinging downstream drift, $\bar{\lambda}$ increases with increasing $\bar{u}_f^a$ (red markers).

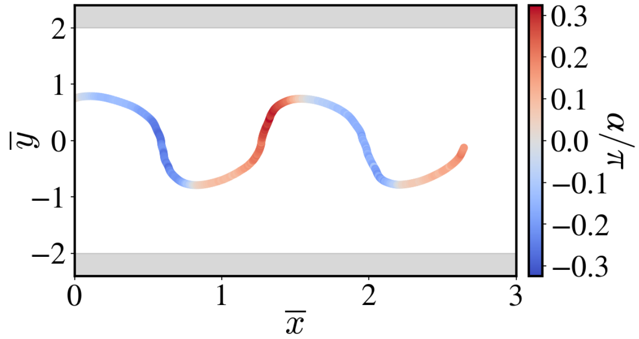

Supplemental Figure S2: Variation in the offset angle α between the intrinsic swimming orientation of the active droplet ($\hat{e}$) and the rheotactic (translational) direction over the oscillatory trajectory for $\bar{u}_f^a = 0.42$.

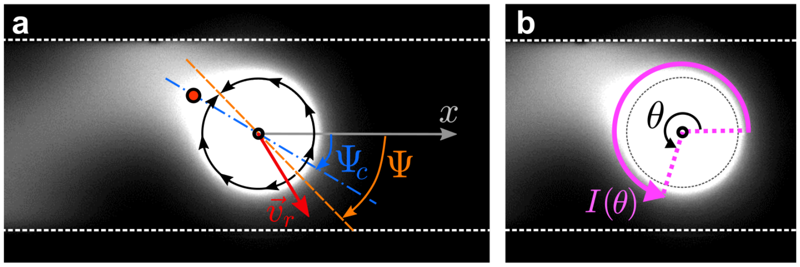

Supplemental Figure S3: In the main manuscript, we use a back-calculation from the measured flow field to infer the orientation Ψ, despite having an additional direct measure via the 'chemical angle' Ψ_c, as measured from fluorescent microscopy data. The reason for this is that Ψ_c is systematically underestimated, as follows: The chemical trail of filled micelles is advected downstream by the ambient flow. Consequently, the location of maximum fluorescence intensity, in the vicinity of the rear stagnation point, is always slightly displaced to the left (right), relative to the upstream swimming direction in the $X - Y$ plane, when the droplet is oriented in a positive (negative) sense relative to $+\hat{x}$. So, we always obtain a relatively smaller angle for Ψ_c compared to Ψ (panel (a), with the difference between angles exaggerated for the sake of clarity). Therefore, Ψ is a more reliable estimate of the intrinsic swimming direction than Ψ_c — however, Ψ and Ψ_c show similar trend and are also quantitatively comparable, if not identical. Furthermore, this exercise also proves that the filled micelle trail is not distorted during rheotaxis to trigger secondary interactions (see ref. 24) with the swimming droplet, and thereby interfering with the rheotactic dynamics.

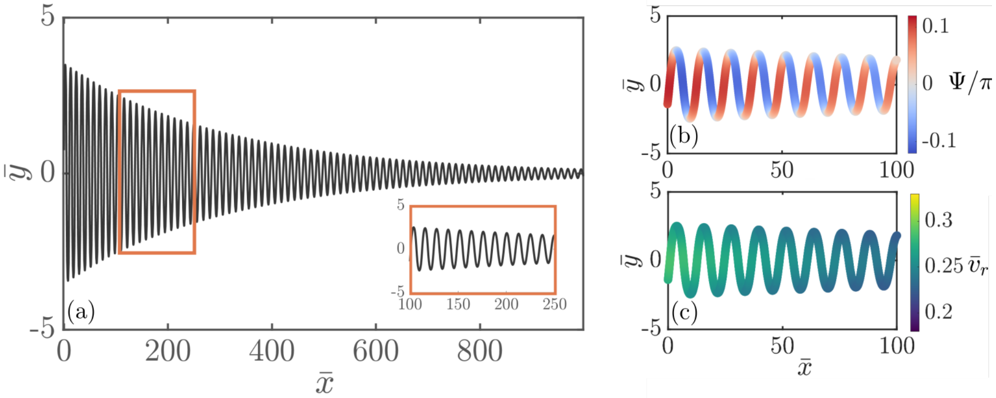

Supplemental Figure S4: While our droplet experiments are limited to weak pushers, we can generalise the predictions of the theory model to other hydrodynamic swimmer classes and compare to existing literature. We demonstrate this here for the rheotactic trajectory of a finite-sized, puller-type microswimmer in a microchannel, as predicted by our theoretical model for $\alpha = -0.22$, $\beta = 0.26$, $\gamma = -0.08$, and $\bar{u}_f^a = 0.42$. (a) The puller-type microswimmer gradually migrates to the channel centre as it swims upstream in an oscillatory trajectory. Similar migration of puller-type microswimmers during rheotaxis has been experimentally observed very recently for *Chlamydomonas* [2]. (b) As the microswimmer migrates towards the channel centre during rheotaxis, its orientation relative to the axial direction, Ψ,

gradually reduces. At the channel centre line, the microswimmer eventually becomes parallel to the axial direction. (c) The translational/rheotactic velocity of the puller-type microswimmer also reduces as it migrates towards the channel centre. Eventually, at the channel centre line, the microswimmer attains a steady-state velocity. The chosen values of the microswimmer size ($R_d \sim 10\ \mu$m), and intrinsic swimming velocity ($v_0 \sim 70\ \mu$m$\cdot$ s^{-1}) are estimated from [2].

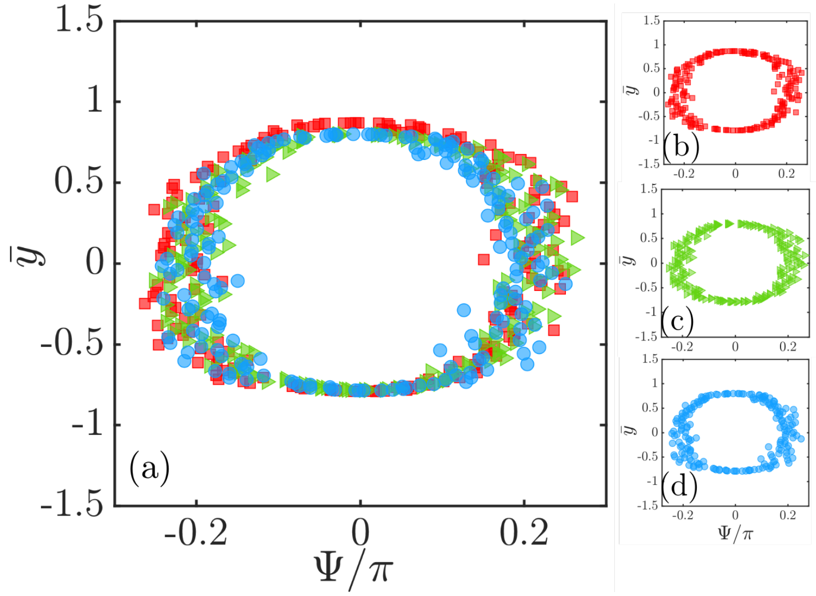

Supplemental Figure S5: Additional experimental data sets showing stable limit cycles for the oscillatory upstream rheotaxis of the droplet microswimmer at comparable values of $\bar{u}^a_f$ (~ 0.4). In (a), the different markers/colours represent the different experimental data sets for a similar imposed flow rate. For clarity, the different data sets are also shown separately in (b)-(d). We have recorded approximately 7 limit cycles for a definite flow rate.

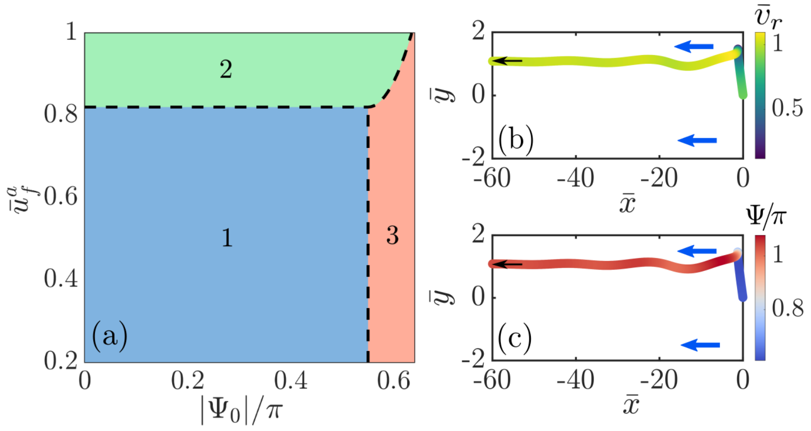

Supplemental Figure S6: (a) Phase diagram showing three different states for the active droplet over the $\bar{u}^a_f$-$|\Psi_0|$ parameter space. The dotted lines showing the boundaries between the different states are qualitative in nature, obtained from a coarse sweep of the parameter space in numerics. Note that due to the strong wall adhesion of active droplets in a quiescent medium we were effectively limited to the cases $\Psi_0 = 0$ and $\Psi_0 = \pi$ in experiments.

Here, Ψ_0 is the initial orientation of the droplet at the instant of actuation of the external flow ($t = 0$). Considering that the imposed flow is always along $-\hat{x}$, $|\Psi_0|/\pi < 0.5$ implies that the droplet is initially upstream oriented. States 1 and 2 represent the oscillatory upstream rheotaxis and the swinging downstream drift respectively, which are discussed in detail in the main text. State 3 represents a downstream sliding of the active droplet along the microchannel wall for an initially downstream oriented droplet ($|\Psi_0|/\pi > 0.55$) (also see Supplemental Movie S4). For higher values of $\bar{u}^a_f$, the behaviour of the active droplet eventually transitions from state 3 to state 2. However, the threshold $\bar{u}^a_f$ necessary for this is relatively larger than that required for the transition from state 1 to state 2.
(b) The active droplet trajectory colour coded by $\bar{v}_r$, and (c) by the intrinsic orientation (Ψ), during the downstream

sliding along the microchannel wall (State 3). (b) and (c) are obtained using Eqs. 3-5 for the initial conditions- $\bar{y}_0(\bar{t} = 0) = 0.014$, $\Psi_0/\pi = 0.62$, and $\bar{u}_f^a = 0.42$. Note that for $\bar{u}_f^a = 0.42$ and $|\Psi_0|/\pi < 0.55$ we get the oscillatory upstream rheotaxis (state 1) as discussed in the main text. State 3, and more generally the dynamics for $|\Psi_0|/\pi > 0.55$, needs to be investigated further in future studies.

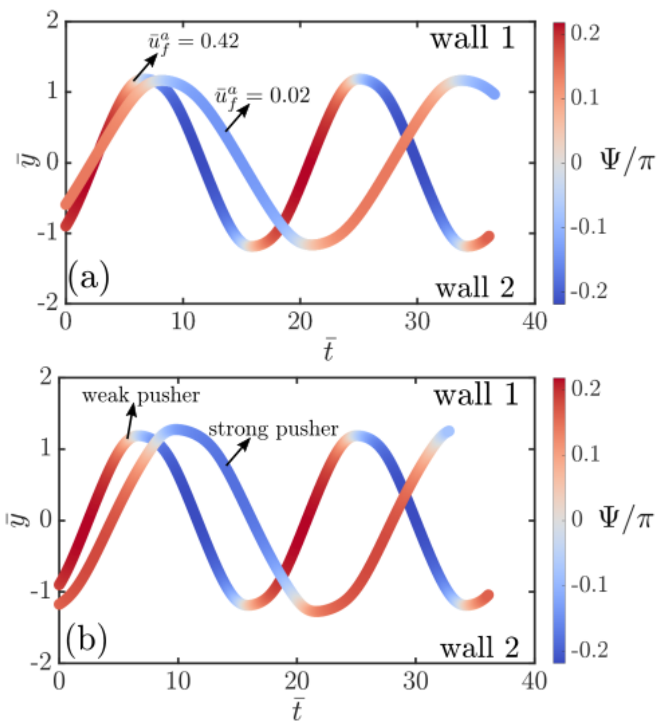

Supplemental Figure S7: Physically consistent estimates for the residence times of microswimmers adjacent to the walls during rheotaxis can be made using our model. Rough estimates for the residence time can be made using the temporal variations in the transverse location (y) of the microswimmer relative to the walls and in its intrinsic swimming orientation (Ψ), as shown in panels (a) and (b). Simply put, a microswimmer spending more time adjacent to a wall with a small magnitude of the orientation angle Ψ can be considered to have a longer residence time. In this spirit, one can qualitatively conclude that the residence time of the active droplet microswimmer decreases with increasing value of the imposed flow (panel a). Furthermore, for the same value of the imposed flow, a strong pusher has a longer residence time compared to a finite-sized, weak pusher kind of a microswimmer.

III. VECTORIAL FORMS OF THE IMAGE SYSTEMS

Following [1], the vectorial forms of the velocity fields due to the image systems for the force-dipole and source-dipole singularities corresponding to *wall 2* (see Fig. 7 in the main text) can be written as

$$\begin{aligned}
&\boldsymbol{u}^*_{fd}\left(\boldsymbol{r}-\boldsymbol{r}^*_0;\hat{e}(\Psi)\right)\\
&=\cos^2\Psi\Bigg[\frac{\Delta\boldsymbol{r}^*}{\Delta r^{*3}}-\frac{3\left(\hat{x}\cdot\Delta\boldsymbol{r}^*\right)^2\Delta\boldsymbol{r}^*}{\Delta r^{*5}}+2l\Bigg\{-\frac{\hat{y}}{\Delta r^{*3}}+\frac{15\left(\hat{x}\cdot\Delta\boldsymbol{r}^*\right)^2\left(\hat{y}\cdot\Delta\boldsymbol{r}^*\right)\Delta\boldsymbol{r}^*}{\Delta r^{*7}}-\frac{3\left(\hat{y}\cdot\Delta\boldsymbol{r}^*\right)\Delta\boldsymbol{r}^*}{\Delta r^{*5}}\\
&-\frac{6\left(\hat{x}\cdot\Delta\boldsymbol{r}^*\right)\left(\hat{y}\cdot\Delta\boldsymbol{r}^*\right)\hat{x}}{\Delta r^{*5}}+\frac{3\left(\hat{x}\cdot\Delta\boldsymbol{r}^*\right)^2\hat{y}}{\Delta r^{*5}}\Bigg\}-2l^2\Bigg\{-\frac{3\Delta\boldsymbol{r}^*}{\Delta r^{*5}}-\frac{6\left(\hat{x}\cdot\Delta\boldsymbol{r}^*\right)\hat{x}}{\Delta r^{*5}}+\frac{15\left(\hat{x}\cdot\Delta\boldsymbol{r}^*\right)^2\boldsymbol{r}^*}{\Delta r^{*7}}\Bigg\}\Bigg]\\
&+\sin^2\Psi\Bigg[\frac{\Delta\boldsymbol{r}^*}{\Delta r^{*3}}-\frac{3\left(\hat{y}\cdot\Delta\boldsymbol{r}^*\right)^2\Delta\boldsymbol{r}^*}{\Delta r^{*5}}+4l\Bigg\{-\frac{\hat{y}}{\Delta r^{*3}}+\frac{3\left(\hat{y}\cdot\Delta\boldsymbol{r}^*\right)\Delta\boldsymbol{r}^*}{\Delta r^{*5}}\Bigg\}+2l\Bigg\{\frac{\hat{y}}{\Delta r^{*3}}+\frac{15\left(\hat{y}\cdot\Delta\boldsymbol{r}^*\right)^3\Delta\boldsymbol{r}^*}{\Delta r^{*7}}\\
&-\frac{9\left(\hat{y}\cdot\Delta\boldsymbol{r}^*\right)\Delta\boldsymbol{r}^*}{\Delta r^{*5}}-\frac{3\left(\hat{y}\cdot\Delta\boldsymbol{r}^*\right)^2\hat{y}}{\Delta r^{*5}}\Bigg\}-2l^2\Bigg\{-\frac{3\Delta\boldsymbol{r}^*}{\Delta r^{*5}}-\frac{6\left(\hat{y}\cdot\Delta\boldsymbol{r}^*\right)\hat{y}}{\Delta r^{*5}}+\frac{15\left(\hat{y}\cdot\Delta\boldsymbol{r}^*\right)^2\boldsymbol{r}^*}{\Delta r^{*7}}\Bigg\}\Bigg]\\
&+0.5\sin2\Psi\Bigg[\frac{6\left(\hat{x}\cdot\Delta\boldsymbol{r}^*\right)\left(\hat{y}\cdot\Delta\boldsymbol{r}^*\right)\Delta\boldsymbol{r}^*}{\Delta r^{*5}}-4l\Bigg\{-\frac{\hat{x}}{\Delta r^{*3}}+\frac{3\left(\hat{x}\cdot\Delta\boldsymbol{r}^*\right)\Delta\boldsymbol{r}^*}{\Delta r^{*5}}\Bigg\}-4l\Bigg\{\frac{\hat{x}}{\Delta r^{*3}}\\
&+\frac{15\left(\hat{x}\cdot\Delta\boldsymbol{r}^*\right)\left(\hat{y}\cdot\Delta\boldsymbol{r}^*\right)^2\Delta\boldsymbol{r}^*}{\Delta r^{*7}}-\frac{3\left(\hat{x}\cdot\Delta\boldsymbol{r}^*\right)\Delta\boldsymbol{r}^*}{\Delta r^{*5}}-\frac{3\left(\hat{y}\cdot\Delta\boldsymbol{r}^*\right)^2\hat{x}}{\Delta r^{*5}}\Bigg\}+4l^2\Bigg\{\frac{15\left(\hat{y}\cdot\Delta\boldsymbol{r}^*\right)\left(\hat{x}\cdot\Delta\boldsymbol{r}^*\right)\Delta\boldsymbol{r}^*}{\Delta r^{*7}}\\
&-\frac{3\left(\hat{y}\cdot\Delta\boldsymbol{r}^*\right)\hat{x}}{\Delta r^{*5}}-\frac{3\left(\hat{x}\cdot\Delta\boldsymbol{r}^*\right)\hat{y}}{\Delta r^{*5}}\Bigg\}\Bigg]
\end{aligned}\tag{1}$$

$$\begin{aligned}
&\boldsymbol{u}^*_{sd}\left(\boldsymbol{r}-\boldsymbol{r}^*_0;\hat{e}(\Psi)\right)\\
&=\cos\Psi\Bigg[-\frac{\hat{x}}{\Delta r^{*3}}+\frac{3\left(\hat{x}\cdot\Delta\boldsymbol{r}^*\right)\Delta\boldsymbol{r}^*}{\Delta r^{*5}}-2l\Bigg\{\frac{15\left(\hat{x}\cdot\Delta\boldsymbol{r}^*\right)\left(\hat{y}\cdot\Delta\boldsymbol{r}^*\right)\Delta\boldsymbol{r}^*}{\Delta r^{*7}}-\frac{3\left(\hat{x}\cdot\Delta\boldsymbol{r}^*\right)\hat{y}}{\Delta r^{*5}}\\
&-\frac{3\left(\hat{y}\cdot\Delta\boldsymbol{r}^*\right)\hat{x}}{\Delta r^{*5}}\Bigg\}+2\Bigg\{\frac{\hat{x}}{\Delta r^{*3}}+\frac{15\left(\hat{x}\cdot\Delta\boldsymbol{r}^*\right)\left(\hat{y}\cdot\Delta\boldsymbol{r}^*\right)^2\Delta\boldsymbol{r}^*}{\Delta r^{*7}}-\frac{3\left(\hat{x}\cdot\Delta\boldsymbol{r}^*\right)\Delta\boldsymbol{r}^*}{\Delta r^{*5}}\\
&-\frac{3\left(\hat{y}\cdot\Delta\boldsymbol{r}^*\right)^2\hat{x}}{\Delta r^{*5}}\Bigg\}\Bigg]+\sin\Psi\Bigg[\frac{3\hat{y}}{\Delta r^{*3}}-\frac{9\left(\hat{y}\cdot\Delta\boldsymbol{r}^*\right)\Delta\boldsymbol{r}^*}{\Delta r^{*5}}+2l\Bigg\{\frac{15\left(\hat{y}\cdot\Delta\boldsymbol{r}^*\right)^2\Delta\boldsymbol{r}^*}{\Delta r^{*7}}\\
&-\frac{6\left(\hat{y}\cdot\Delta\boldsymbol{r}^*\right)\hat{y}}{\Delta r^{*5}}-\frac{3\Delta\boldsymbol{r}^*}{\Delta r^{*5}}\Bigg\}-2\Bigg\{\frac{\hat{y}}{\Delta r^{*3}}+\frac{15\left(\hat{y}\cdot\Delta\boldsymbol{r}^*\right)^3\Delta\boldsymbol{r}^*}{\Delta r^{*7}}-\frac{9\left(\hat{y}\cdot\Delta\boldsymbol{r}^*\right)\Delta\boldsymbol{r}^*}{\Delta r^{*5}}\\
&-\frac{3\left(\hat{y}\cdot\Delta\boldsymbol{r}^*\right)^2\hat{y}}{\Delta r^{*5}}\Bigg\}\Bigg]
\end{aligned}\tag{2}$$

Here, $\Delta\boldsymbol{r}^*=\boldsymbol{r}-\boldsymbol{r}^*_0$, where $\boldsymbol{r}^*_0$ is the location of the image system corresponding to the wall, and $\Delta r^*=|\Delta\boldsymbol{r}^*|$. For *wall 2*, $\boldsymbol{r}^*_0=\boldsymbol{r}_0-2(\bar{y}_0+\bar{w})\hat{y}=\boldsymbol{r}_0-2l\hat{y}$. Subsequently, the velocity field due to the image system for the source quadrupole singularity, corresponding to *wall 2*, is computed as [1]

$$\begin{aligned}
\left[\boldsymbol{u}^*_{sq}\left(\boldsymbol{r}-\boldsymbol{r}^*_0;\hat{e}(\Psi)\right)\right]&=\left[\nabla|_{\boldsymbol{r}_0}\boldsymbol{u}^*_{sd}\right][\hat{e}]\\
&=\left[\begin{pmatrix}\frac{\partial}{\partial\bar{x}_0}\\ \frac{\partial}{\partial\bar{y}_0}\end{pmatrix}\begin{pmatrix}u^*_{sd,x} & u^*_{sd,y}\end{pmatrix}\right]\begin{bmatrix}\cos\Psi\\ \sin\Psi\end{bmatrix}
\end{aligned}\tag{3}$$

Note here that the ∇ operator acts on the co-ordinates of the squirmer centroid location.

SUPPLEMENTARY REFERENCES

[1] S. E. Spagnolie and E. Lauga, Hydrodynamics of self-propulsion near a boundary: Predictions and accuracy of far-field approximations, *Journal of Fluid Mechanics* **700**, 105–147 (2012)

[2] T. Omori et al., Rheotaxis and migration of an unsteady microswimmer, *Journal of Fluid Mechanics*, **930**, A30 (2022)
