# Supplementary material for: Oscillatory rheotaxis of artificial swimmers in microchannels
Source: Nat Commun. 2022 May 26;13:2952. doi: 10.1038/s41467-022-30611-1 (PMC9135748; doi:10.1038/s41467-022-30611-1)
Supplement: Supplementary file 3 — Description of additional Supplementary File [file 41467_2022_30611_MOESM3_ESM.pdf]

Descriptions of Additional Supplementary Data files

Movie 1: Active droplets in parallel channels without imposed flow. The droplets adhere to the channel side walls.

Movie 2 : Control of rheotactic displacement from upstream oscillation over in-place trapping to downstream drift for a droplet by regulating the imposed flow.

Movie 3: Example of two nearly synchronously oscillating droplets under similar flow conditions in two parallel channels. Initially, strong flow sweeps the droplets downstream at the walls; later, under weaker flow, and guided around corners, they are made to simultaneously travel upstream or are trapped in place by controlling the imposed flow.

Movie 4: The rheotactic behaviour depends on the initial conditions. If the imposed flow starts in the droplet's swimming direction, the droplet will not switch direction to perform upstream oscillation, even if the flow speed would permit it - as in this case, where the imposed flow is below the critical threshold for upstream swimming.
